# Wood Moisture-Content Measurement Accuracy of Impregnated and Nonimpregnated Wood

**DOI:** 10.3390/s21217033

**Published:** 2021-10-23

**Authors:** Jacek Barański, Aleksandra Suchta, Sylwia Barańska, Ivan Klement, Tatiana Vilkovská, Peter Vilkovský

**Affiliations:** 1 Energy Institute, Faculty of Mechanical Engineering and Ship Technology, Gdańsk University of Technology, Narutowicza 11/12, 80-233 Gdańsk, Poland; 2 Institute of Machine and Materials Technology, Faculty of Mechanical Engineering and Ship Technology, Gdańsk University of Technology, Narutowicza 11/12, 80-233 Gdańsk, Poland; aleksandra.konopka@pg.edu.pl; 3 Department of Molecular Genetics of Bacteria, Faculty of Biology, Gdańsk University, Wita Stwosza 59, 80-308 Gdańsk, Poland; sylwia.baranska@ug.edu.pl; 4 Department of Wood Technology, Faculty of Wood Sciences and Technology, Technical University in Zvolen, T. G. Masaryka 24, 96001 Zvolen, Slovakia; klement@tuzvo.sk (I.K.); t.hurakova21@gmail.com (T.V.); peter.vilkovsky@tuzvo.sk (P.V.)

**Keywords:** wood drying, impregnated wood, pine wood, wood moisture content, wood resistance, moisture content, moisture meter resistance

## Abstract

The influence of the impregnation process of pine wood (*Pinus sylvestris* L.) samples on the electrical resistance changes and the moisture-content measurement accuracy is presented in this paper. In this study, the resistances of impregnated and nonimpregnated green pine timber harvested from northern Poland were compared. An impregnation method based on a vacuum-pressure chamber was used. Copper salts were applied as the impregnated solutions. The obtained results of the electrical resistance comparison showed a dependence of wood resistance on the moisture content. Higher conductivity occurred in impregnated wood samples filled with copper salt compared with wood samples without impregnation. Noticeable differences in the electrical resistance values were observed when the wood moisture content was significantly above the Fibre Saturation Point (FSP).

## 1. Introduction

The moisture content (MC) of green timber varies between 40% and 70%, and it needs to be decreased to around 10–15%. Therefore, it is necessary to dry it to a balanced level. In order to optimise the drying process and to achieve the target MC, it is important to have systems which are able to measure or predict the changes in MC during drying and final wood treatment. Requirements from timber buyers and from new drying standards are increasingly focusing on the importance of obtaining the correct final MC.

The drying process impacts deformations, surface checking, discoloration, and hence the product quality and the manufacturing costs. A good drying process may prevent the timber from developing outer and inner cracks as well as several other defects. It increases the timber strength; nails, screws and glue hold better, paint and finishes adhere well. This process is a function of time which is influenced by many different parameters such as temperature, diffusion coefficient (wood structure), dimension of wood, drying medium speed, its relative humidity and other factors. Many of these parameters can be controlled during the drying process with reliable sensors. However, controlling the moisture flow throughout the whole piece of wood is not possible yet. On a timber stack level, the inhomogeneity of the initial MC and the natural scatter of moisture-related wood properties pose a challenge to the operator monitoring the drying process and achieving the required drying quality. Research in this field is of great importance for the wood industry, as the industrial drying process always needs to be continuously improved as market demands increase and new wood products are developed.

The most important quality factor in the chain production and the final wood products’ application is the wood MC [1]. Reliable methods for determining MC are essential, both for the producer and the user, in order to avoid the problems that can occur when timber is used with an inappropriate MC. Industrial tests of commercial online MC meters have shown low accuracy of individual readings [2,3]. All MC measuring methods have their advantages and disadvantages, and most of today’s meters only use one measuring technique [4]. Some subjective methods, such as estimating MC from smell, weight, or the way the wood behaves, have been used in the past by experienced people. The results made by these methods have been compared with reliable measurements and it has been clearly shown that the first ones are unreliable. These methods can provide rough data, but the inherent errors are far too great for them to be used for proper wood quality control.

Examples of moisture sensors for wood and wood-based materials are widely applied, employing a variety of sensing technologies, including, for example, electrical resistance [5,6,7], acoustics [8,9], microwaves [10,11], and near-infrared spectroscopy (NIR) [12,13]. Dielectric sensors have also been successfully applied using, for example, radio frequency spectroscopy [14], time or frequency domain reflectometry [15,16], and direct capacity measurements [17,18,19]. Each of these approaches has its own merits, but most of them are not entirely practical for flowing streams of material, which is required in industrial applications of process control. Additionally, the accuracy of wood MC measurements can be improved by taking into account the visual properties [3].

The electrical resistance of wood decreases with increasing MC [20] and a common method in wood MC determination is the electrical resistance measurement between two electrodes inserted in the wood. The relationship between MC and material resistance is different in various MC ranges. It is affected by the species wood, experimental variables and calibration experiments. The measurement result is influenced by many factors, e.g., temperature, the content of extractives, etc. Capacitive moisture meters consider the density of the wood, which can differ significantly even inside a single board. The reliability of wood MC readings with electrical resistance meters decreases with an increase of MC above 20% and below 9%. In dry wood up to 7% MC, the reduction in electrical conductivity is about five-fold for an increase of 1% in MC [21]. From 7% MC to the Fibre Saturation Point (FSP), the decrease in resistivity is slightly smaller, about two to four times for every 1% increase in MC [20]. Above the FSP, the resistance decreases with MC increase, but compared to the changes in electrical conductivity below fibre saturation, the changes are small [22].

All MC measurement systems have their limitations. Nowadays, for sawn timber kilns, there are no commercial monitoring systems for MC measurement where each single board of the stack can be monitored.

On other hand, the kiln drying is accomplished by controlling the equilibrium moisture content (EMC) which depends on the drying medium’s humidity and temperature in the drying kiln. To make the process faster, the EMC should be lower and vice versa. This can be achieved by automatic (computerized) simultaneous control of heater, fan, and ventilation. While the drying process of the timber surface is directly controlled by setting the EMC, drying of the inside material highly depends on the surface properties, its state, and also the type of timber, velocity, and humidity of drying medium inside the kiln, hence it is not easy to control [23].

The increased use of wood in its natural form, as a construction material and as a renewable and low-embodied energy, is an alternative to reinforced concrete and steel. In a certain environments and applications, issues related to durability, fire resistance and dimensional stability need to be solved [24,25,26]. In general, the treatment of wood through chemical and thermal modifications, coatings or impregnation offers effective ways to address some of these issues [27]. In particular, “controlled” impregnation of specific monomers into the wood cell cavity (lumen) and also into the wood cell wall [28,29,30] followed by polymerization, can enhance the performance of wood in construction by improving its mechanical properties, giving the wood higher durability and fire resistance [28,31,32,33,34].

This article presents the results of the electrical resistance changes and thus MC measurement accuracy in selected samples impregnated and nonimpregnated of pine wood (*Pinus sylverstis* L.). The obtained results of the electrical resistance comparison showed a dependence of wood resistance on the moisture content. It has been observed that a higher conductivity occurred in impregnated wood samples filled by copper salt than wood samples without impregnation. Noticeable differences in the electrical resistance values were observed when the wood moisture content was significantly above the Fibre Saturation Point (FSP). The applied water-soluble impregnate is an aqueous copper salt solution that penetrates on a capillary and diffusion basis, and the MC of impregnated wood does not significantly impact its penetration into the material. The diffusion intensity is directly proportional to the impregnation salt’s aqueous (water)-solution concentration and depends on the duration of this phenomenon. It continues after removing the wood sample from the salt solution until the wood is dried and the wood MC reaches a value below the fibre saturation point (FSP). The impregnation method based on the vacuum–pressure chamber was used.

## 2. Materials and Methods

The material used during the experiments was pine wood (*Pinus sylvestris* L.). The wood for the impregnated wood samples (three boards) was initially dried in industrial conditions until the MC was below FSP. Then, they were full-scale impregnated in an autoclave. The impregnation process continued for 120 min, and the retention level was 1.0 dm^3^/(m^3^.min).

**Figure 1:**
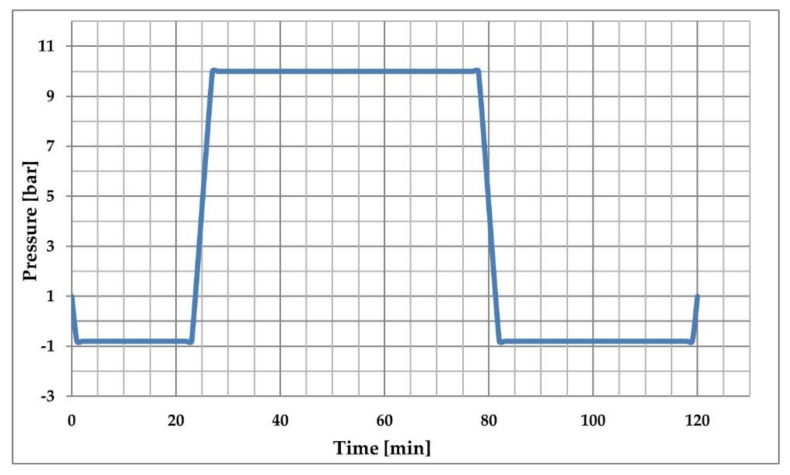
The course of phases or pressure changes of the impregnation process preformed in the autoclave.

The so called full-cell impregnation method is based on the technique widely described in detail by Babiński (1992) [35]. The boards were placed in the impregnation solution environment under atmospheric pressure. The first impregnation phase lasted 25 min in a vacuum of −0.8 bar. In the next step, a pressure of 10 bar was maintained for 55 min. After the second impregnation phase when the pressure was decreased to atmospheric, the surplus of impregnation solution was removed from the autoclave. The final phase, the impregnation step, during which the impregnation water solution is sucked out of the lumens, was carried out under a pressure of −0.8 bar and lasted 40 min. The pressure changes in time during the impregnation process are presented in Figure 1. A preservative (TANALITH E3475, Arch Timber Protection, Castleford, UK) and colouring (TANATONE 3950, Arch Timber Protection, Castleford, UK) agents based on copper salt were used. Tanalith E3474 contains basic copper carbonate (copper(II), carbonate-copper(II), hydroxide(1:1)): 15.7% *w*/*w* pure substance or 9% *w*/*w* expressed in copper. The concentration of impregnate solution was 3.8%. The other three wood boards, which were not impregnated, were freshly cut.

There are also other preservatives, including coal-tar substances such as creosote, oil-based chemicals such as pentachlorophenol (PCP), and aqueous solutions of compounds such as chromated copper arsenate (CCA), ammoniacal copper zinc arsenate (ACZA), and copper azole (CA-B). An example of a CA-B preservative is TANALITH E3475. Creosote, PCP and CCA are used on heavy structural members such as railroad ties, utility poles, marine poles, and bridge timbers, while ACZA and CA-B are used on common structural timber. The impregnating solution contains salts, such as copper (III) carbonate and copper hydroxide. In addition, it also contains 2-aminoethanol (NH_3_CH_2_CH_2_OH) alcohol and organic acids. As a result of the reaction of 2-aminoethanol with organic acids, salts are formed.

**Figure 2:**
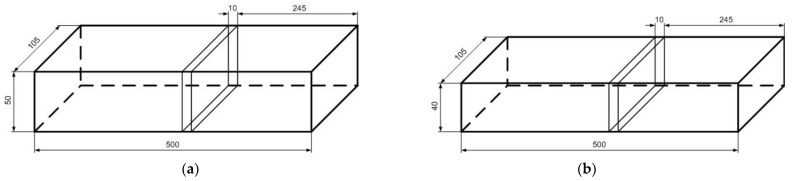
Dimensions of wood samples prepared for an experiment: (**a**) nonimpregnated wood, (**b**) impregnated wood. The wood samples taken for the initial wood MC determination (applying the gravimetric method).

**Figure 3:**
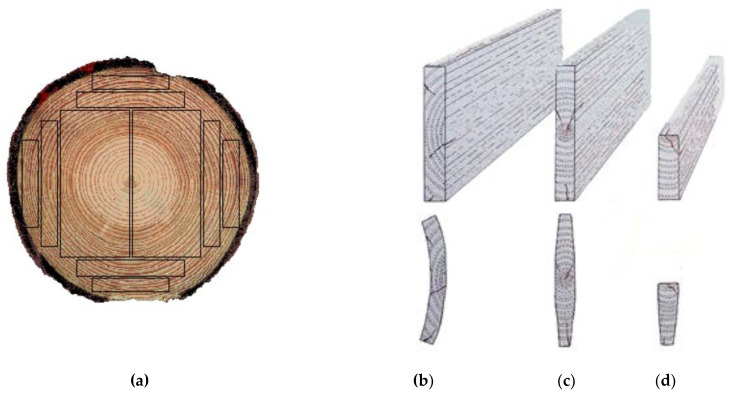
Types of growth-ring orientations (**a**) within the obtained boards: (**b**) tangential, (**c**) axial, (**d**) radial.

Before the experiments, the wood was prepared as 500 mm-long boards (Figure 2). The growth rings of this wood were tangential (Figure 3a,b). The wood that was intended for impregnation process was cut into pieces (samples) of the following dimensions: 120 mm × 105 mm × 40 mm (Figure 2b). The other boards (nonimpregnated before experiments) were also cut into pieces, but the dimensions were as follows: 60 mm × 105 mm × 50 mm, respectively (Figure 2a) [36,37,38,39,40].

The wood for the research was obtained from Sylva Ltd. Co. sawmill in Wiele, Poland. Wood samples without heartwood were selected. Values of basic properties such as initial and final MC and density of impregnated and nonimpregnated pine wood are presented in Table 1. These properties and the salt concentration in wood are very important with respect to electrical resistance measurement.

**Figure 4:**
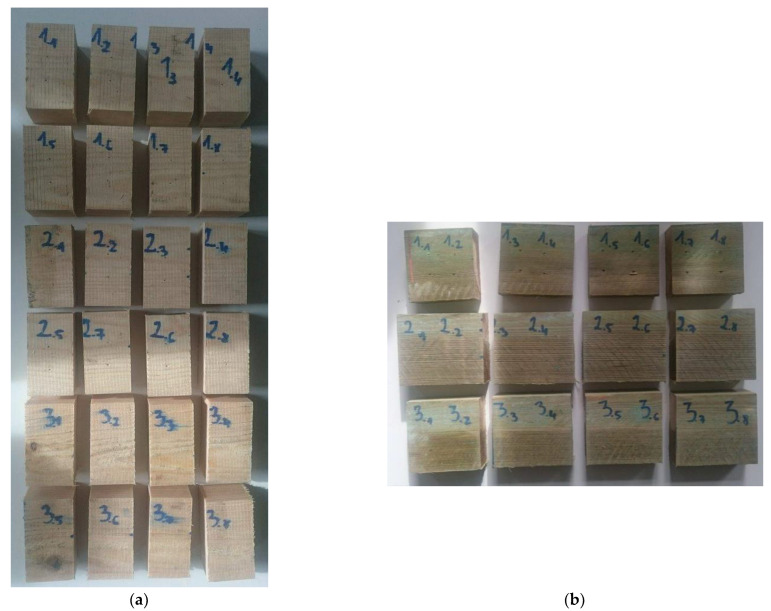
The view of wood samples prepared for experiments: (**a**) nonimpregnated, (**b**) impregnated.

Each pine wood sample was seasoned in open-air conditions. The measurements were performed at 24 h intervals in the laboratory, with conditions at 25 °C and a relative humidity ϕ of 29.5%. For these parameters, the equilibrium MC was *W_r_* = 6%. The drying time was about 30 days for impregnated wood and about 45 days for nonimpregnated wood (Figure 4).

The gravimetric method was used to determine wood MC. The samples were taken from the centre of the 500 mm boards (Figure 2). This method is more accurate than the commonly used methods with MC sensors based on resistance measurement. The test stand was equipped with a precision balance to measure the mass of the samples. The mass measurements were made with an accuracy of 0.001 g. The drying process of samples to an oven-dry state was performed in the laboratory kiln at 103 ± 2 °C. MC was calculated using Equation (1):

(1) $$MC_{g}=\frac{m_{w}-m_{0}}{m_{w}}\cdot 100\%$$

where:

*m_w_* is the mass of the moisture sample [grams];
*m_o_* is the mass of the absolute dry sample [grams].

Then, the wood MC was measured using an electrical-resistance moisture meter Hydromette type RTU 600 (Gann Mess-u. Regeltechnik GmbH, Gerlingen, Germany). The moisture meter was calibrated for a room temperature of 25 °C and for the specified wood species: Scots pine. The MC measuring range was 4–100% [41].

**Figure 5:**
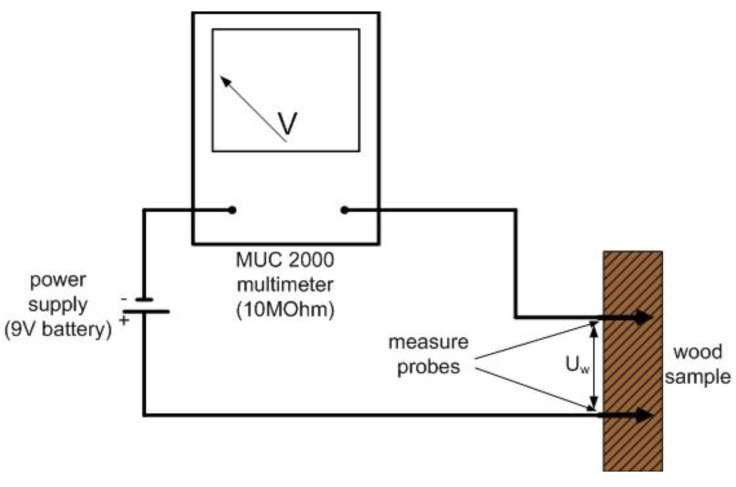
Schematic diagram of wood-resistance measurement system.

To determine the resistance of impregnated and nonimpregnated pine wood samples, the measuring system was used (Figure 5). It consisted of an MUC 2000 multimeter (Slandi, Michalowice, Poland) with an internal resistance of 10 MΩ, a power supply generating a constant voltage of 9.45 V [42], and measurement probes within the Hydromette RTU 600 moisture meter. The probes were placed at the same measuring points in a sapwood.

The resistance of the test samples was determined with the following general formula:

(2) $$I=\frac{U}{R}=\frac{U_{s}}{R_{m}+R_{w}}=\frac{U_{m}}{R_{m}}=\frac{U_{w}}{R_{w}}$$

The constant voltage *U_s_* was calculated using equation presented below:

(3) $$U_{s}=U_{m}+U_{w}$$

The resistance of the pine wood *R_w_* was calculated with the following equation:

(4) $$R_{w}=R_{m}\cdot \;\left(\frac{U_{s}}{U_{m}}-1\right)$$

where:

*U_s_* is the constant voltage generated by power supply [9.45 V];
*R_m_* is the internal resistance of the multimeter, [10 MΩ];
*U_m_* is the voltage indicated by multimeter [V];
*U*_w_ is the voltage of wood samples [V] and
*R*_w_ is the resistance of the pine wood [MΩ].

## 3. Results and Discussion

**Figure 6:**
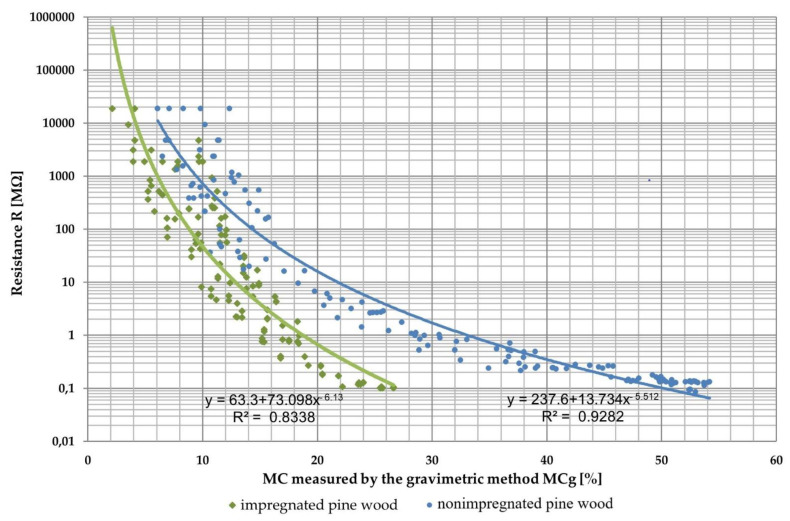
Resistance characteristics of impregnated and nonimpregnated pine wood.

The experiment examined pine wood resistance as a function of its MC; 24 samples of nonimpregnated boards and 24 samples of impregnated boards were tested. The resistance curves differed for impregnated wood and nonimpregnated boards due to differences in resistance values of the tested wood. The characteristic resistance points of studied wood were approximated with an exponential function (Figure 6). The results imply that electrical resistance drops more rapidly and then more and more gradually with increasing MC. In these regression curves, the coefficient of determination, R^2^, is very high and is equal to 0.8338 for impregnated and 0.9282 for nonimpregnated wood. The deviation of the measured resistance values near the regression curves is significant due to the large variation in the electrical properties of wood. The deviation decreases with higher wood MC.

**Figure 7:**
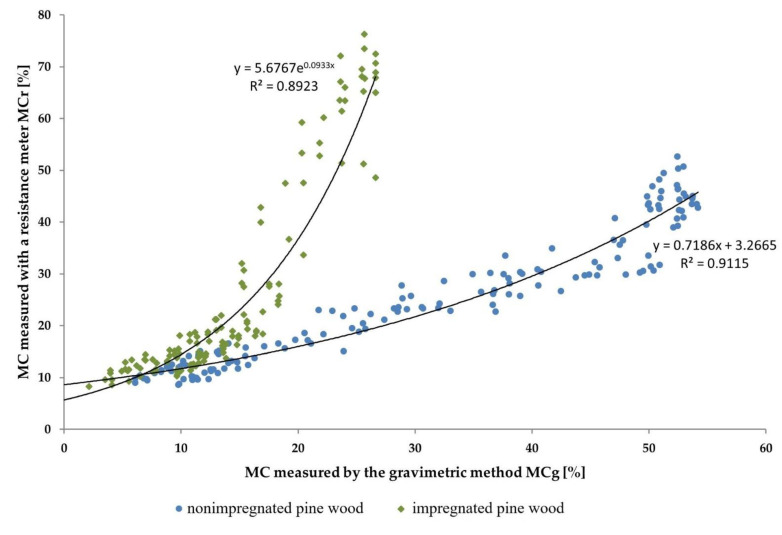
Measurement differences of the pine wood MC as a result of the wood impregnation process.

Then, the resistance moisture meter was used to determine the impact of wood impregnation on the error of measuring its MC. The reference values of MC were obtained using the gravimetric method with a 0.001 g accuracy balance. The results from the measurements are shown in Figure 7. The measurement of nonimpregnated wood MC using the resistance meter was in good agreement with the gravimetric method. This is because there were no chemical additives that could change the resistance of the dried material. However, impregnated wood MC values using the resistance meter were consistent with the gravimetric method only when it was below 20%. In such wood samples there was only a small amount of water in the material, so the chemical additives did not influence overall wood resistance. Above 20% MC, there were very big differences between resistance meter and gravimetric method measurements. This is because the wood samples contained a mixture of water together with the chemical additives, and this mixture affects wood’s electrical resistance.

The results of MC measurement of nonimpregnated wood with a resistance meter are characterized by a slight deviation from the reference values measured by the gravimetric method up to the FSP level. As the MC increased above the FSP, the error during measurement was higher, which is in accordance with the information in the resistance meter manufacturer’s manual data. In the case of this measurement for the impregnated wood, the deviation increased exponentially above the values of MC equal to 15% (measured by gravimetric method). Above this value, the use of an appropriate correction formula was necessary.

**Figure 8:**
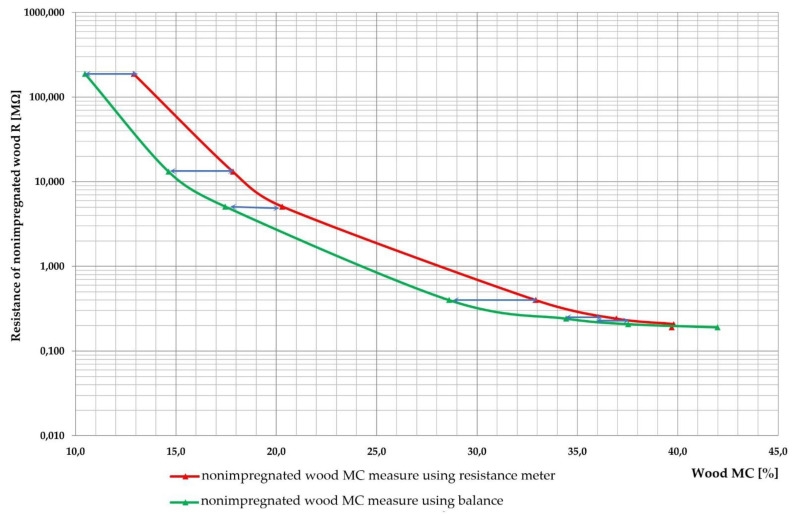
Measurement differences of the nonimpregnated pine wood MC as a result of the moisture-content measurement method.

**Figure 9:**
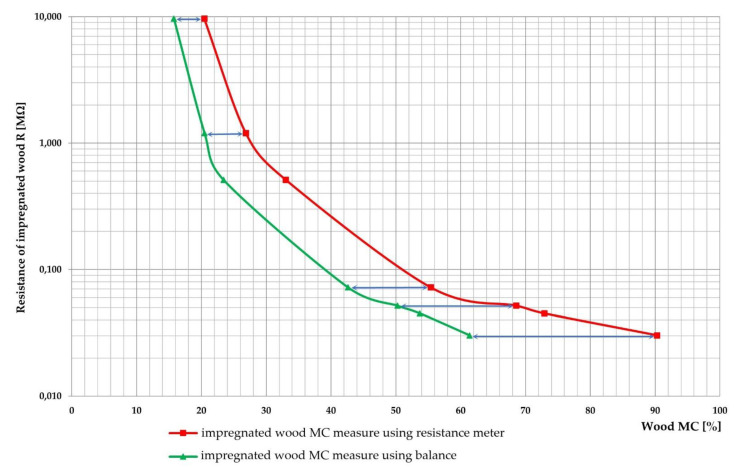
Measurement differences of the impregnated pine wood moisture content (MC) as a result of the moisture-content measurement method.

After determining the impact of wood impregnation on the MC measurement error using the resistance moisture meter, the differences in wood MC of nonimpregnated and impregnated wood were compared (Table 2) and graphically presented (Figure 8 and Figure 9), and the value of wood MC difference at constant wood resistance was calculated using the formula presented below:

(5) $$\Delta_{MC}={\left[\frac{\left(MC_{g}-MC_{r}\right)}{MC_{g}}\right]}^{2}\cdot 100$$

where:

*MC_g_* is the wood MC using gravimetric method [%];
*MC_r_* is the wood MC using resistance meter, [%].

**Table 2:** Differences of MC of nonimpregnated and impregnated pine wood using gravimetric and resistance meter methods at constant resistance.

	Nonimpregnated Wood			Impregnated Wood		
	Wood Samples’ Resistance	Gravimetric Method	Resistance Meter Method	Wood Samples’ Resistance	Gravimetric Method	Resistance Meter Method
		MC	MC		MC	MC
	[MΩ]	[%]	[%]	[MΩ]	[%]	[%]
1.	0.190	41.99	39.70	0.030	61.40	90.30
2.	0.208	37.53	39.80	0.045	53.70	72.90
3.	0.241	34.44	36.90	0.052	50.20	68.60
4.	0.399	28.60	32.90	0.072	42.60	55.40
5.	5.101	17.45	20.30	0.511	23.40	33.00
6.	13.158	14.63	17.80	1.196	20.50	26.90
7.	188.380	10.47	12.90	9.632	15.80	20.50

The results of the calculations according to Formula 5 are presented in Table 3. It can be noticed that moisture-content differences for nonimpregnated boards increase from 2.290 to 23.209% proportionally with wood resistance changes from 0.19 to 188.380 MΩ. For impregnated wood, the MC differences vary between 47.068 and 29.746% with wood resistance changes from 0.03 to 9.632 MΩ. For impregnated wood samples, the resistance changes are higher than for nonimpregnated ones. On the other hand, moisture-content differences for both types of pine wood samples are similar, at about 13.25% for nonimpregnated boards and around 17.6% for impregnated wood.

The statistical analyses were performed using Statistica 13.1 software with the alpha level set at 0.0.5. To check the influence of the moisture-measuring method and the wood-impregnation process on the moisture-content values; the analysis of covariance (ANCOVA) was applied. Statistical analyses showed that both wood impregnation (F_1,301_ = 707.9; *p* < 0.001) and method of moisture measuring (F_1;301_ = 90.229; *p* < 0.001) significantly influence the moisture content throughout the time of the experiment (F_4;301_ = 528.45; *p* < 0.001).

**Figure 10:**
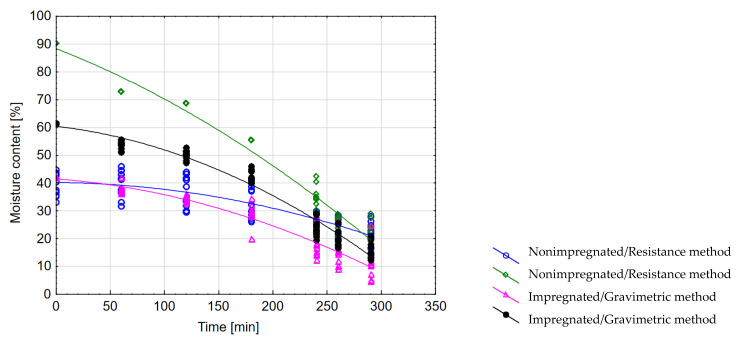
The influence of wood impregnation process and the measurement method on the wood moisture-content value.

**Figure 11:**
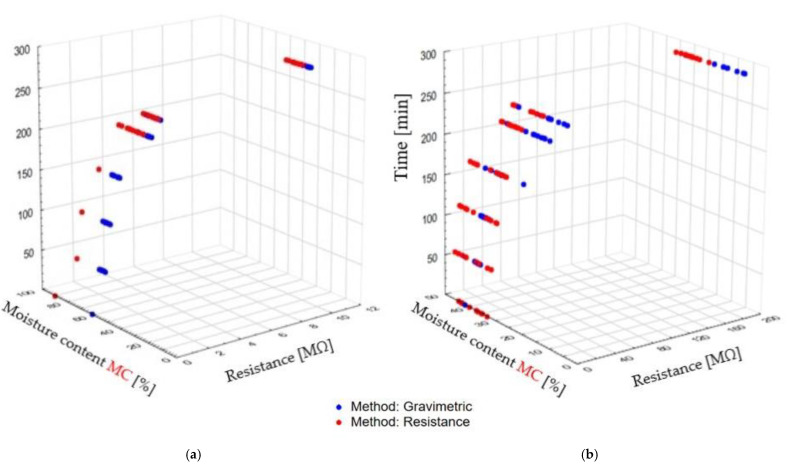
The wood impregnation process impact on the moisture content and resistance values during drying processes: (**a**) impregnated wood, (**b**) nonimpregnated green timber.

**Figure 12:**
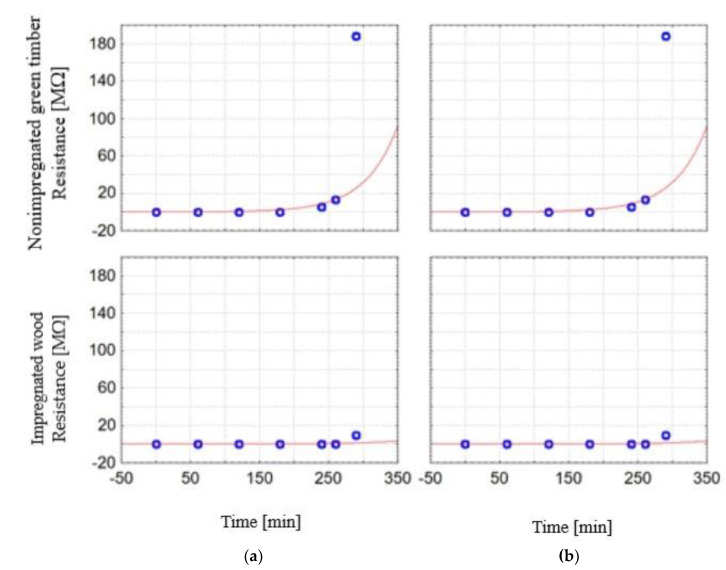
The measurement method impact on the wood resistance during drying process: (**a**) resistance method, (**b**) gravimetric method.

The results of this task are presented in Figure 10, Figure 11 and Figure 12. Pine wood impregnation preceding the drying process leads to an increase in the average MC compared with the average MC obtained with nonimpregnated timber measured at the same time intervals (analysis of covariance (ANCOVA) F_4;304_ = 623.95; *p* < 0.001), as shown in Figure 10. Differences in the curve courses indicates that MC measurement during drying depends on the wood impregnation process and the measurement method.

The gravimetric moisture-content measurement method indicates a lower MC value of dried material, both of impregnated and nonimpregnated wood, compared to the resistance moisture-content measurement method. Nonimpregnated wood is characterized by lower moisture-content values regardless of its measurement method.

It should also be added that both the impregnation process and the time of drying process significantly affect the material resistance values, as seen in Figure 12. At the beginning of the drying process, for about 250 min, the resistance of nonimpregnated timber and impregnated wood is low, in the ranges 0.19–13.158 MΩ and 0.03–1.196 MΩ, respectively, and in a short period of time it rapidly increases to values of 188.38 MΩ and 9.632 MΩ for nonimpregnated and impregnated wood, respectively. The resistance differences between the nonimpregnated and impregnated woods vary from 60 to 200%. This is the effect of the moisture removal process during drying in both wood types, and the retention of copper-based salts in the impregnated wood.

## 4. Conclusions

This work presents the results of wood MC measurement accuracy in impregnated wood and nonimpregnated boards.

The impregnation process of pine wood (*Pinus sylvestris* L.) impacts the resistance values and thus the accuracy of MC measurements. Impregnation of wood with the preservatives and colouring agents, TANALITH E3475 and TANATONE 3950, respectively, lowered the electrical resistance and consequently increased the apparent measured MC that was predicted with the moisture meter (Hydromette RTU 600) at the default calibration settings.

The MC measurements of impregnated pine wood using a resistance meter were significantly different from MC measurements using the gravimetric method. Such a phenomenon was particularly noticeable above the FSP level.

The resistance MC measurement method is not suitable for MC measurement of impregnated pine wood when the MC content is above 20%. The application of this method requires correction formulas, which would need to be estimated empirically depending on the type and amount of impregnant substance in the wood material.

The wood MC during drying depends on the wood impregnation process and the measurement method. The gravimetric measurement method indicates a lower value of dried material MC, both in impregnated and nonimpregnated wood, compared with the resistance MC measurement method. The wood impregnation and the time of the drying process significantly influence the material’s resistance values. The resistance differences between the nonimpregnated and impregnated woods vary over a wide range. This is a result of moisture removal during the drying process in both wood types, and the retention of copper-based salts in the impregnated wood.

The coefficient of determination, R^2^, for nonimpregnated green timber was higher than for the impregnated wood based on separate data-fitting equations. The obtained results corresponding to impregnated wood were better fitted using an exponential rather than a linear function.

Pine wood impregnation preceding the drying process leads to an increase in the average MC compared with the average MC obtained with nonimpregnated timber measured at the same time intervals.

## Figures and Tables

**Table 1:** Values of initial and final MC and density (ρ) of impregnated and nonimpregnated pine wood.

			Impregnated Wood	Nonimpregnated Sawnwood
1.	Average initial MC	MC_i_ [%]	27	55
2.	Average final MC	MC_f_ [%]	6.5	8.6
3.	Average initial density	ρ_i_ [kg/m^3^]	640	520
4.	Average final density	ρ_f_ [kg/m^3^]	530	480

**Table 3:** The moisture-content differences between nonimpregnated and impregnated pine wood using gravimetric and resistance-meter methods.

	Nonimpregnated Boards				Impregnated Boards			
	Wood Samples’ Resistance	Gravimertic Method	Resistance Meter Method	Moisture-Content Difference	Wood Samples’ Resistance	Gravimetric Method	Resistance Meter Method	Moisture-Content Difference
		Moisture Content	Moisture Content			Moisture Content	Moisture Content	
	[MΩ]	[%]	[%]	[%]	[MΩ]	[%]	[%]	[%]
1.	0.190	41.99	39.70	0.297	0.030	61.40	90.30	22.154
2.	0.208	37.53	39.80	0.365	0.045	53.70	72.90	12.783
3.	0.241	34.44	36.90	0.510	0.052	50.20	68.60	13.434
4.	0.399	28.60	32.90	2.260	0.072	42.60	55.40	9.028
5.	5.101	17.45	20.30	2.667	0.511	23.40	33.00	16.831
6.	13.158	14.63	17.80	4.695	1.196	20.50	26.90	9.746
7.	188.380	10.47	12.90	5.386	9.632	15.80	20.50	8.848

## Data Availability

The data presented in this study are available on request from the corresponding author. The data are not publicly available due to copywrite.

## References

[1] Esping B., Salin J.G., Brander P. (2005). Fukt i trä för byggindustrin.

[2] Esping B. (2003). Test av kommersiella fuktkvotsmätare in-line.

[3] Nilsson M. (2010). Evaluation of Three in-Line Wood Moisture Content Meters. Master’s thesis.

[4] Vikberg T. (2010). Fuktkvotsmätare för träindustrin: En kartläggning av metoder för mätning av fuktkvoter i intervallet 7–18 fuktkvotsprocent.

[5] Dai G. (2001). Ahmet, K. Long-term monitoring of timber moisture content below the fiber saturation point using wood resistance sensors. For. Prod. J..

[6] Tamme V., Muiste P., Tamme H. (2013). Experimental study of resistance type wood moisture sensors for monitoring wood drying process above fibre saturation point. For. Stud..

[7] Brischke C. (2014). Lampen, S.C. Resistance based moisture content measurements on native, modified and preservative treated wood. Eur. J. Wood Wood Prod..

[8] Brashaw B.K., Wang X., Ross R.J., Pellerin R.F. (2004). Relationship between stress wave velocities of green and dry veneer. For. Prod. J..

[9] Chan J.M., Walker J.C., Raymond A.A. (2011). Effects of moisture content and temperature on acoustic velocity and dynamic MOE of radiata pine sapwood boards. Wood Sci. Technol..

[10] Lundgren N., Hagman O., Johansson J. (2006). Predicting moisture content and density distribution of Scots pine by microwave scanning of sawn timber II: Evaluation of models generated on a pixel level. J. Wood Sci..

[11] Moschler W.W., Hanson G.R., Gee T.F., Killough S.M., Wilgen J.B. (2007). Microwave moisture measurement system for lumber drying. For. Prod. J..

[12] Jones P.D., Schimleck L.R., Peter G.F., Daniels R.F., Clark A. (2005). Nondestructive estimation of Pinus taeda L. wood properties for samples from a wide range of sites in Georgia. Can. J. For. Res..

[13] Schimleck L.R., Evans R., Matheson A.C. (2009). Estimation of Pinus radiata D. Don clear wood properties by near-infrared spectroscopy. J. Wood Sci..

[14] Tomppo L., Tiitta M., Laakso T., Harju A., Venalainen M., Lappalainen R. (2009). Dielectric spectroscopy of Scots pine. Wood Sci. Technol..

[15] Wullschleger S.D., Hanson P.J., Todd D.E. (1996). Measuring stem water content in four deciduous hardwoods with a time-domain reflectometer. Tree Physiol..

[16] Woodhead I.M., Buchan G.D., Christie J.H., Irie K. (2003). A general dielectric model for time domain reflectometry. Biosyst. Eng..

[17] Jazayeri S., Ahmet K. (2000). Detection of transverse moisture gradients in timber by measurements of capacitance using a multiple-electrode arrangement. For. Prod. J..

[18] Xinguang L., Mingying X. (2009). Applied research on moisture content measurement: One sided capacitance sensors. Meas. Control..

[19] Jensen P.D., Hartmann H., Böhm T., Temmerman M., Rabier F., Morsing M. (2006). Moisture content determination in solid biofuels by dielectric and NIR reflection methods. Biomass Bioenergy.

[20] Stamm A.J. (1927). The electrical resistance of wood as a measure of its moisture content. Ind. Eng. Chem..

[21] Skaar C. (1988). Wood-Water Relations.

[22] Stamm A.J. (1929). The fiber-saturation point of wood as obtained from electrical conductivity measurements. Ind. Eng. Chem. Anal. Ed..

[23] Flotaker S., Tronstad S. (2000). Description and Initial Test of 8 Principles for in-Kiln Measuring and End-Point Control of Wood Moisture Content. http://www.treteknisk.no/resources/filer/publikasjoner/rapporter/Rapport-47.pdf.

[24] Ramage M.H., Burridge H., Busse-Wicher M., Fereday G., Reynolds T., Shah D.U., Wu G., Yu L., Fleming P., Densley-Tingley D. (2017). The wood from the trees: The use of timber in construction. Renew. Sustain. Energy Rev..

[25] Rowell R.M. (2013). Structure and function of wood. Handbook of Wood Chemistry and Wood Composites.

[26] Forsén H., Tarvainen V. (2000). Accuracy and Functionality of Hand-Held Wood Moisture Content Meters.

[27] Hill C.A. (2006). Wood Modification: Chemical.

[28] Militz H. (1993). Treatment of timber with water soluble dimethylol resins to improve their dimensional stability and durability. Wood Sci. Technol..

[29] Schneider M.H. (1995). New cell wall and cell lumen wood polymer composites. Wood Sci. Technol..

[30] Keplinger T., Cabane E., Chanana M., Hass P., Merk V., Gierlinger N., Burgert I. (2015). A versatile starategy for grafting polymers to wood cell walls. Acta Biomater..

[31] Rowell R.M., Konkol P. (1987). Treatments That Enhance Physical Properties of Wood.

[32] Lande S., Westin M., Schneider M. (2004). Properties of furfurylated wood. Scand. J. For. Res..

[33] Marney D., Russell L. (2008). Combined fire retardant and wood preservative treatments for outdoor wood applications–A review of the literature. Fire Technol..

[34] Krzysik F. (1978). Nauka o Drewnie (The science about the wood).

[35] Babiński L.K. (1992). Impregnacja drewna metodą próżniową. Ochr. Zabyt..

[36] EN 1995-1-1:2004 (2004). Eurocode 5: Design of Timber Structures—Part 1-1: General—Common Rules and Rules for Buildings.

[37] EN 13183-1:2002 (2002). Moisture Content of a Piece of Sawn Timber—part 1: Determination by Oven Dry Method.

[38] EN 13183-2:2002 (2002). Moisture Content of a Piece of Sawn Timber—Part 2: Estimation by Electrical Resistance Method.

[39] EN 13183-3:2005 (2005). Moisture Content of a Piece of Sawn Timber—Part 3: Estimation by Capacitance Method.

[40] ISO 13061-1: 2014 Physical and mechanical properties of wood-Test methods for small clear wood specimens-Part 1: Determination of Moisture Content for Physical and Mechanical Tests. https://www.iso.org/standard/60063.html.

[41] Gann Mess- u. Regeltechnik GmbH, Gerlingen, Germany, RTU 600 an Electronic Wood Moisture Meter Data. http://www.gann.de/Produkte/ElektronischeFeuchtigkeitsmessgeräte/ClassicSerie/HydrometteRTU600/tabid/104/lang.

[42] Slandi Ltd. Co Michalowice, Poland, MUC 2000 Digital Multimeter Data. http://polskiemultimetry.prv.pl.

